# Effects of some anesthetic agents on skin microcirculation evaluated by laser Doppler perfusion imaging in mice

**DOI:** 10.1186/1746-6148-9-255

**Published:** 2013-12-17

**Authors:** Sara Gargiulo, Matteo Gramanzini, Raffaele Liuzzi, Adelaide Greco, Arturo Brunetti, Giancarlo Vesce

**Affiliations:** 1 Institute of Biostructures and Bioimages of the National Council of Research, Via T. De Amicis 95, Naples 80145, Italy; 2 Department of Advanced Biomedical Sciences, University of Naples Federico II, Via Pansini 5, Naples 80145, Italy; 3 CEINGE scarl, Via G. Salvatore 486, Naples 80145, Italy; 4 Department of Veterinary Medicine and Animal Productions, University of Naples Federico II, Via Delpino 1, Naples 80137, Italy

**Keywords:** Microvascular perfusion, Anesthesia, Murine model, Laser Doppler perfusion imaging

## Abstract

**Background:**

Anesthetic agents alter microcirculation, influencing tissue oxygenation and delivery of vital substrates. Laser Doppler perfusion imaging is a widespread technique in the field of microvascular research that can evaluate noninvasively and in real time the effects of environmental conditions, physical manipulations, diseases and treatments on peripheral perfusion. This study aims to evaluate laser Doppler perfusion imaging as a means to detect changes in skin microcirculation induced by some popular anesthetic agents in a murine model. Twenty-four age- and gender-matched healthy CD1 mice were examined by laser Doppler perfusion imaging. The skin microcirculatory response was measured at the level of plantar surfaces during isoflurane anesthesia with or without subsequent dexmedetomidine or acepromazine. At the end of the procedure, dexmedetomidine was reversed by atipamezole administration.

**Results:**

In all mice, skin blood flow under isoflurane anesthesia did not show significant differences over time (P = 0.1). The serial perfusion pattern and values following acepromazine or dexmedetomidine administration differed significantly (P < 0.05).

**Conclusions:**

We standardized a reliable laser Doppler perfusion imaging protocol to non-invasively assess changes in skin microcirculation induced by anesthesia in mice, considering the advantages and drawbacks of this technique and its translational value.

## Background

Microcirculation is the final link between the cardiovascular system and cellular interfaces and, ultimately, molecular processes. Many studies have investigated the effect of anesthetics on peripheral and systemic microcirculation in humans [1-5], especially their effects on microvascular perfusion, aiming to ensure adequate tissue oxygenation and nutritional supply. Mice are an ideal model to study anesthetic action due to their easy manipulation, well-established behavioral and homeostatic responses to anesthesia, and well-known genetic background. Outbred mouse strains are widely used in toxicology and pharmacology, and CD1 mice have been employed in anesthesia research [6-8] on the assumption that most characteristics of interest have a polygenic inheritance and are related to phenotypic variation in a genetically heterogeneous population [9,10]. Moreover, anesthesia is required for most in vivo studies using mouse microcirculatory models, and the use of diverse anesthetic agents in translational research can interfere with experimental results [11,12]. As an example, pentobarbital [13,14], midazolam-medetomidine [15] and isoflurane [16] have been used in preclinical studies on peripheral arterial disease to evaluate the effects of new angiogenetic therapies. Microcirculatory responses to the most popular inhalation (halothane, isoflurane) or injectable anesthetics (propofol-fentanyl, barbiturates and ketamine) have been investigated in rats at the level of intestinal [17], cremaster or dorsal muscle microcirculation [18-20] using invasive dorsal microcirculatory chambers or intravital microscopy. So far, few data have been reported regarding the microvascular effects of the popular laboratory-animal anesthetic agents acepromazine and dexmedetomidine. Acetylpromazine maleate is an α-adrenergic receptor antagonist broadly used for sedation and balanced anesthesia in animals [21]. Concurrent administration of acepromazine reduces the required dose of isoflurane while potentiating peripheral vasodilation and lowering blood pressure in dogs [22]. The combination of acepromazine with ketamine and xylazine is recommended for a safe and reliable surgical anesthesia in mice, although it is associated with marked hypotension [23,24]. Dexmedetomidine hydrochloride is a selective α_2_-adrenoceptor agonist with preferential affinity for α_2_A and α_2_B receptors [21]. Perioperative administration of dexmedetomidine hydrochloride reduces the required doses of isoflurane, thiopental and propofol in humans and animals, and it reduces the activation of the sympathetic nervous system during surgery, preventing harmful hemodynamic events such as acute kidney injury [25]. Reliable techniques for measuring perfusion in accessible tissues such as skin may have significant potential to improve our understanding of microvasculature regulation under anesthesia. Laser Doppler perfusion imaging (LDPI) is a noninvasive technique allowing real-time quantification of skin perfusion in two-dimensional color-coded images. Enhancement of the measured area provides a better evaluation of blood flow heterogeneity, allowing for the identification of subtle changes in skin perfusion induced by anesthesia and indicating circulatory status in other areas [26,27]. Although LDPI offers a simple and accurate estimate of peripheral perfusion, a standard method for the study of microcirculatory changes related to anesthesia in mice is lacking. In the present study, we reviewed several biological variables, such as gender, environmental variables and operational variables, such as body temperature, skin district and recording conditions, to develop a LDPI protocol to evaluate the effects of some anesthetic agents on microcirculation in mice. Our LDPI protocol is a potentially valuable research tool to detect in vivo real-time microcirculatory changes in preclinical experiments in mice.

## Results

**Figure 1:**
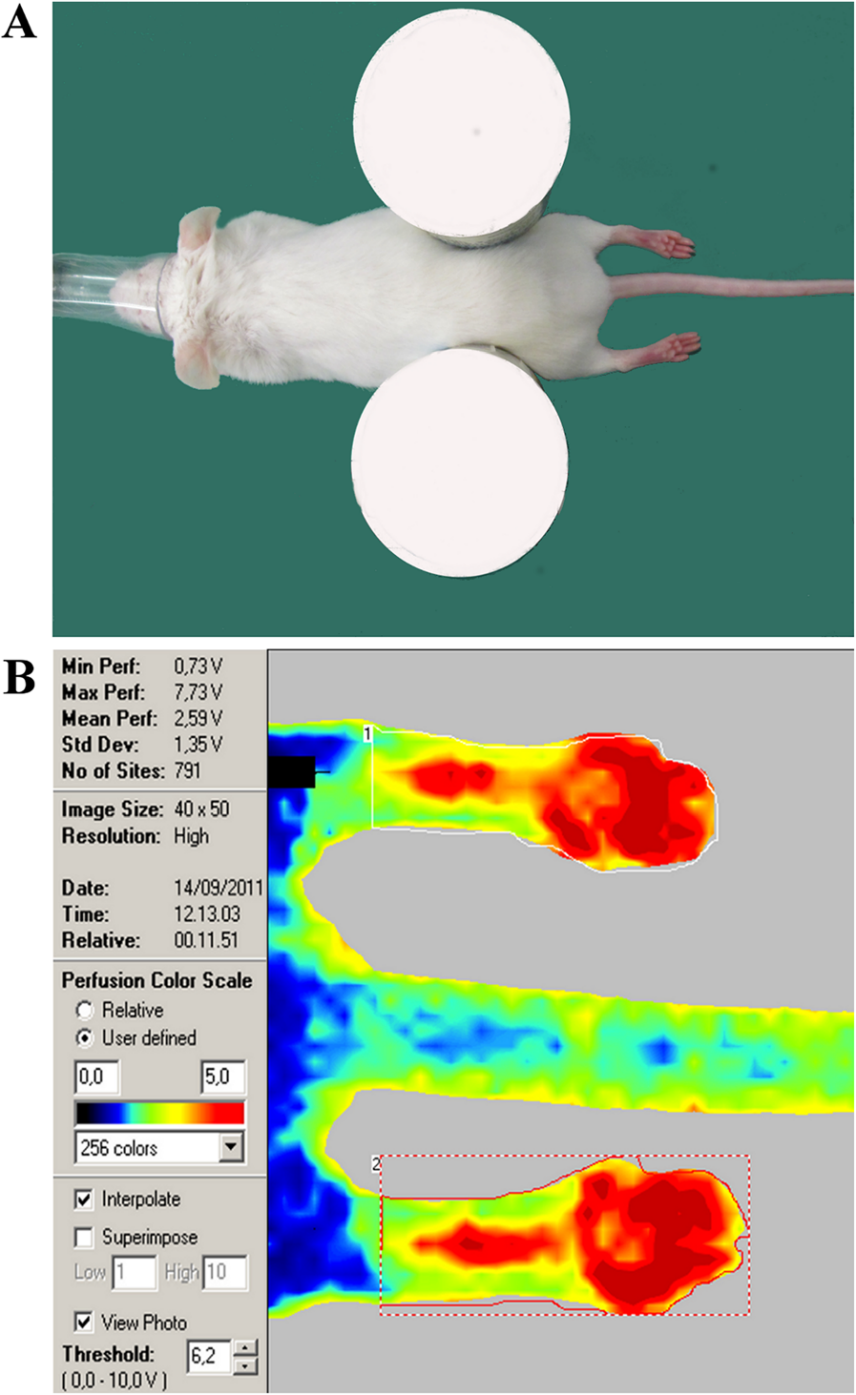
**LDPI scan technique. (A)** Animal positioning in sternal recumbency on a light-absorbing pad, with the hind plantar surfaces symmetrical and perpendicular to the laser beam. **(B)** LDPI image post-processing and measurement standardized protocol: the mean intensity of the Doppler signal was registered in ROI encompassing the hind paws and expressed as numerical value normalized for their area (perfusion color scale 0–5 volts).

**Figure 2:**
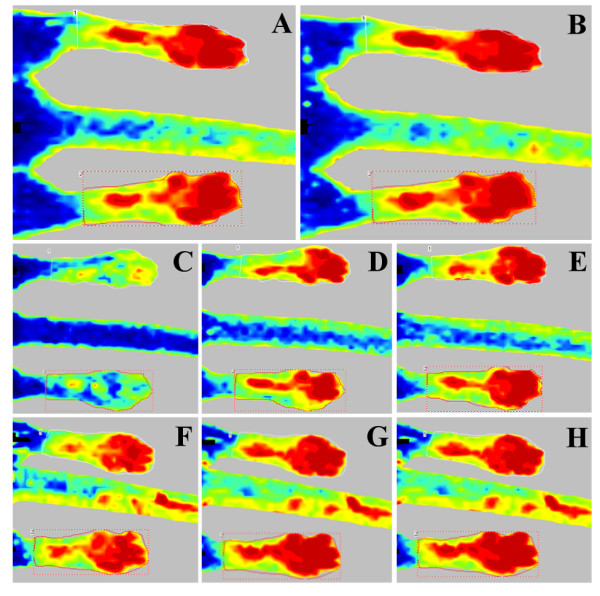
**Representative LDPI images.** Peripheral perfusion patterns in mice over time after administration of 3 anesthetic protocols. Time points with significant differences (P < 0.05) are reported. Group 1 (top row) 10 **(A)** and 20 minutes after acepromazine injection **(B)**; group 2 (middle row) 5 **(C)** and 15 minutes after dexmedetomidine injection **(D)** and 5 minutes after atipamezole administration **(E)**; control group (lower row) 10 **(F)**, 20 **(G)** and 30 minutes after isoflurane maintenance **(H)** (perfusion color scale 0–5 volts as reported in Figure 1).

Standardized protocol for animal positioning and LDPI image post-processing and measurement are described in Figure 1. Sequential perfusion units (PU, volts) values for each group are reported in Table 1 as median, minimum and maximum values. No significant differences were seen between males’ and females’ peripheral blood flow (PBF) at any time point (P > 0.05). The effects of different anesthetics on peripheral perfusion for each group are presented in Figure 2. In all mice, mean perfusion under isoflurane anesthesia showed an increasing trend at 10 and 20 minutes after maintenance (4.25 to 4.55 volts), reaching a steady perfusion value without significant differences among groups and in later times (P = 0.1). In contrast, the mean LDPI values following acepromazine (group 1) and dexmedetomidine administration (group 2) differed significantly. Between 10 and 20 minutes after acepromazine administration, a significant perfusion increase (P = 0.005) was observed, from 4.55 to 4.85 volts. Dexmedetomidine administration produced a clear biphasic effect, leading to a significantly reduced (P = 0.0001) blood perfusion (2.47 volts) after 5 minutes, followed by an increase to 4.32 volts (P = 0.008) after 15 minutes. The latter perfusion value, close to that under isoflurane anesthesia (P = 0.6), was quite retained (4.34 volts) even following dexmedetomidine reversal by atipamezole (the antidote to the α_2_-receptor agonist) (P = 0.9). No significant peripheral perfusion changes were observed in control mice after up to 30 minutes (4.57 volts) of 1.5% isoflurane anesthesia (P = 0.11).

**Table 1:** Microvascular perfusion values

**Groups**	**Measurement time**					
	***10 min***	***20 min***	***25 min***	***30 min***	***35 min***	***40 min***
*1*	4.37/3.73-4.90	4.57/3.67-5.52		4.53/3.98-5.53		4.84/4.29-5.76
*2*	4.32/3.69-4.75	4.52/3.62-5.33	2.37/2.02-3.13		4.32/3.73-4.81	4.41/3.72-4.72
*3*	4.34/3.71-4.83	4.52/3.66-5.50		4.57/3.85-5.78		

Mouse peripheral PU (volts) in the isoflurane + acepromazine (1), isoflurane + dexmedetomidine (2) and isoflurane-alone (3) experimental groups at significative time points (median/min-max).

## Discussion

Anesthetics modulate microcirculation mainly via autonomic sympathetic and parasympathetic nerves on vascular smooth muscle. Phenothiazine tranquilizers as well as α_2_-agonists exert their hemodynamic effects mainly by interacting with α-adrenergic-receptors. Phenothiazines cause vasodilation predominantly by blocking α_1_ receptors but are also dopamine receptor antagonists [28]. While D_1_-like dopamine receptors induce relaxation of resistance arteries [29,30] D_2_-like dopamine receptors are typically present on postganglionic sympathetic neurons, where their excitation leads to a reduction of the neural release of norepinephrine, inducing a passive fall in vascular resistance and heart rate [31]. Dexmedetomidine is a selective α_2_-adrenoceptor agonist that shows a dose-dependent, preferential affinity for α_2_A and α_2_B receptors [21], evoking a biphasic blood pressure response: a short hypertensive phase mediated by the α_2_B receptors, followed by hypotension mediated by the α_2_A receptors [32,33]. The peripheral hemodynamic effects of phenothiazines and of α_2_-agonists thus differ: while acepromazine causes significant hypotension in isoflurane-anesthetized animals [34], dexmedetomidine [35,36] increases peripheral vascular tone, counteracting the isoflurane-induced vasodilation and reduction in arterial blood pressure [22]. LDPI permits a noninvasive, real-time measurement of microvascular blood flow using two-dimensional color-coded images of skin perfusion. The use of laser doppler flowmetry technique to detect the sympathetic tone during general anesthesia in humans has been reported [3,37,38], and translational approaches using LDPI in microvascular perfusion mouse models offer the advantages of being easy and fast [39]. Moreover, various anesthetics alter blood flow in rodents [40-43], so anesthetic regimens used in mouse microcirculatory models should be taken into account, and they should not adversely affect the vascular bed to be examined. The main finding in this study is that LDPI is able to evaluate in real time the anesthesia-induced changes in mouse peripheral microcirculation. The hemodynamic effects recorded during the different anesthetic protocols were as expected based on previous clinical and animal studies, although the analyzing techniques were different [2-5,22]. Because LDPI scans are disrupted by motion [44] and single or combined sedatives have lacked restraining effects in mice, we chose to perform our study under 1.5% isoflurane anesthesia to record reference perfusion values. Isoflurane produces only minor effects on murine hemodynamic status [6,7]. Costantinides et al. (2011) [45] reported that 1.5% isoflurane produces stable body temperature, mean arterial pressure (MAP) and heart rate (HR) values in mice, comparable to those observed in awake animals, so they recommended it for physiological and pharmacological studies of cardiac function and to facilitate translational research in non-invasive imaging platforms. In the present study, isoflurane anesthesia yielded a reproducible and stable effect on peripheral blood perfusion over time (P = 0.1). Acepromazine increased isoflurane plantar perfusion, as reported by Lemke et al. [22], and reduced vascular tone and arterial pressure due to its α-blocking action. After dexmedetomidine administration, a rapid and intense decrease in plantar perfusion was followed by a longer phase of increased perfusion, in agreement with the typical biphasic hemodynamic effect of this class of sedatives. In all of the mice, the increased perfusion recorded 15 minutes after dexmedetomidine administration did not surpass the perfusion brought about by isoflurane (P = 0.6), and it was noticeably lower than the perfusion recorded following acepromazine administration (P = 0.01), which did not increase significantly even after atipamezole injection (P = 0.9). Special care was taken to avoid methodological bias. To date, the skin has been used as a model of microcirculation to investigate vascular mechanisms in cardiovascular [46-48] or kidney diseases [49] and diabetes [50,51]. Autonomic innervation of microvessels in the region of interest [3,28,52-54], somatic stimulation of cutaneous arterial sympathetic nerve activity [55], positioning and body temperature [56] are all crucial factors affecting skin blood flow measured by LDPI. Glabrous skin areas are highly innervated by noradrenergic sympathetic vasoconstrictor nerves [3,28], which are regulated by α-adrenoceptors [57] in several species [52-54]. For these reasons, we chose plantar region to investigate blood flow changes brought about by anesthetic drugs, also avoiding hair clipping, which might affect cutaneous arterial sympathetic nerve activity and alter LDPI measurements [55]. In our setting, precise hind plantar surface positioning was achieved to keep the site of interest highly symmetrical and precisely perpendicular to the laser beam (Figure 1) [58-60]. Moreover, body temperature was monitored by a rectal probe and adjusted between 35.5-36.5°C by an infrared lamp. Our experiments were performed in a temperature-controlled room [29], and we started LDPI recordings after each animal had acclimatized. The effects of sex hormones on vascular tone continue to be a matter of debate [61,62]. Stucker et al. (2001) [63] reported in an LDPI study only a tendency toward higher perfusion values in men than in women, stating that moderate gender differences in skin perfusion between study groups should be tolerated. Similarly, Kunkel et al. (2007) [64] found that foot skin perfusion in normal human subjects was independent of gender. In our experience, no significant differences between males and females were found in the peripheral blood flow in either control or treated animals. In accordance with the manufacturers’ technical instructions, the room lighting should be kept to a minimum brightness. We set our lighting discrimination between background and the site of measurement to the default threshold level of 6.2 volts, adjusting the backscattered laser light intensity in the range of 7–9 volts, obtaining an optimum quality of data. To compare perfusion in different images, a user-defined color scale was adopted during the acquisition process, ranging from 0 to 5 volts (perfusion output value of 0 volts was calibrated to 0% perfusion, whereas 5 volts was calibrated to 100%). The average perfusion in each region of interest (ROI) was normalized to the wall plantar surface area to reduce bias related to unavoidable anatomical and position variance. To further minimize any data divergence, the hind paw perfusion value for each animal at each time point was calculated as the average value of both hind paw ROIs.

## Conclusions

LDPI is able to evaluate noninvasively and in real time the skin microcirculation changes induced by general anesthesia in mouse models. LDPI could be useful for studying the effects of anesthetics on peripheral microcirculation and to avoid the inconsistent use of anesthetic agents in cardiovascular translational research. Standardization of an appropriate LDPI procedure is needed in preclinical studies to avoid bias in experimental results.

## Methods

### Ethical permission

This study was approved by the animal welfare regulation committee (CESA) of the University “Federico II” of Naples and by the Italian Ministry of Health. It complied with the Guide for the Care and Use of Laboratory Animals published by the US National Institutes of Health (NIH Publication No. 85–23, revised 1996).

### Study subjects and design

Twenty-four CD1 mice (15 females and 9 males), 8 to 10 weeks old, were randomly assigned to one of three experimental groups (5 females and 3 males) and sequentially examined in identical ambient conditions. Skin perfusion was recorded by LDPI under isoflurane anesthesia combined or not with acepromazine or dexmedetomidine, as well as after the administration of atipamezole to antagonize dexmedetomidine’s effects.

### Experimental protocol

Animals were acclimated for 15 min at a room temperature of 27 ± 3°C before anesthetic induction. During LDPI recording, the ambient lighting was kept at a minimum. Body temperature was monitored by a rectal temperature probe (Harvard Apparatus®, MLT1404) and closely adjusted to 35.5 ± 0.5°C by an infrared lamp kept 60 cm away from the body surface. On the basis of a critical revision of the existing literature, peripheral perfusion was measured at the level of the hairless, highly sympathetic innervated plantar surfaces [3,28]. Animals were placed in sternal recumbency on the light-absorbing pad provided by the apparatus company, positioning the hind plantar surfaces symmetrically and perpendicularly to the laser beam (Figure 1). Isoflurane induction and maintenance were identical for all mice: each animal was weighed on a precision scale and transferred from a holding cage to a small rodent anesthetic chamber (isoflurane 4% in 2 L/min oxygen) (ISOFLURANE-VET®, MERIAL ITALIA S.p.A.®). When deeply anesthetized, animals were placed in sternal recumbency on the recording bed and fitted with a facial mask delivering isoflurane 1.5% in 1 L/min oxygen. LDPI scans were recorded 10 and 20 minutes after isoflurane maintenance. Subsequent group treatments were carried out according to the schedule below, with precise time intervals between the LDPI recordings based on the pharmacodynamics of the different anesthetic agents:

***Group 1*** (8 subjects): Acepromazine (PREQUILLAN®, FATRO S.p.A.®) 5 mg/kg (= 0.99 mg/kcal) was administered intraperitoneally (IP), followed by two LDPI scans at an interval of ten minutes.

***Group 2*** (8 subjects): Dexmedetomidine (DEXDOMITOR®, Pfizer Italia Srl®) 1 mg/kg (= 0.19 mg/kcal) was administered IP, followed by two LDPI scans after 5 and 15 minutes. Finally, dexmedetomidine was reversed by injecting the α_2_-adrenoceptor antagonist atipamezole (ATIPAM, Fatro®) 2.5 mg/kg (= 0.49 mg/kcal) IP, and a further LDPI scan was performed after 5 minutes.

***Group 3*** “control” (8 subjects): an additional LDPI scan was recorded 30 minutes after isoflurane maintenance.

### Laser Doppler imaging system

The Periscan® apparatus displayed the blood perfusion signal both as a numerical PU (volts) and as a color-coded image ranging from dark blue (low perfusion) to bright red (high perfusion). The settings used in the present study were laser beam power = 1 mV; wavelength = 670 nm; pixel size = 0.25 × 0.25 mm^2^; scanner head distance =15 cm; scanning area = 3 × 2 cm^2^; scanning time = 2 minutes.

### Data processing

The mean intensity of the Doppler signal was quantified using proprietary software in a fixed ROI, encompassing the corresponding hind paw regions, normalized for the areas of the hind paws and expressed as numerical values (volts) to reduce the bias related to unavoidable anatomical and position variance. To further minimize any data divergence, the hind paw perfusion value for each animal at each time point was calculated as the average value of both hind paw ROIs.

### Data analysis

Statistical analysis was carried out using the software SPSS 18.0.2. (SPSS, Chicago, IL). To compare inter-group differences, one way Friedman ANOVA was used. A post hoc analysis with Dunn’s test was performed when appropriate. A linear generalized model (LGM) for repeated measurements (two-way ANOVA) was used to assess perfusion patterns at different times within groups. A P value <0.05 was considered statistically significant.

## Abbreviations

HR: Heart rate; IP: Intraperitoneally; LDPI: Laser Doppler perfusion imaging; MAP: Mean arterial pressure; PU: Perfusion units; ROI: Region of interest.

## Competing interests

The authors declare that they have no competing interests.

## Authors’ contributions

SG, MG, AB and GV conceived and designed this study, as well as contribuited to data interpretation and drafted the manuscript. SG, MG, and GV carried out the experiments. AG took part in the data collection. SG and MG analysed and arranged data for statistical analysis. RL performed the statistical analyses. SG and MG made equal contribution to this study and should be considered first authors. All authors read and approved the final manuscript.
